# Atlanta Medical Society Reports

**Published:** 1868

**Authors:** 


### ATLANTA MEDICAL SOCIETY REPORTS.

Atlanta, Ga., November 20th, 1867.

Reports of cases being called for—

*Dr. J. M. Johnson* reported a case of senile gangrene, in which the ankle and instep are involved. Under the use of warm applications, nourishing diet, stimulants, etc., the case is slowly improving, bnt does not exhibit sufficient evidences of improvement to warrant entire confidence in his final recovery The subject is a negro man about sixty-eight years old, and has had good health usually. He had met with a similar case before, in a subject sixty-two years old, and, with the treatment adopted in this, succeeded in preserving the life of the patient, and the limb in a sufficient state of perfection to allow the use of it in walking.

He considered the blood-vessels supplying the foot in a condition unfitting them for the performance of their function, especially that of supplying nutrition, and that the death of the parts involved naturally resulted. Ossification, perhaps more frequently at this period of life than any other cause, leads to the obstruction in the circulation of blood through the arteries, and may be set down as one at least among other causes of senile gangrene.

*Dr. W. F. Westmoreland* thought distinct varieties of circumscribed gangrene are found: that which arises from an over amount of blood in the part, called soft gangrene, and that which results from a deficiency in the circulation, called dry mortification. The latter is sometimes best treated by amputation of the limb.

*Dr. Miller* thought the opinion entertained by members, touching the immediate cause and effects of this disease, and that generally received by the profession, is exactly the opposite of the true condition of things which exists in the production and effects of chronic mortification. It is a physiological fact, that the arrest of nutrition in any part, and the consequent arrest of capillary circulation of that part, destroys its vitality, and that the obstruction of the circulation in an artery supplying such part is also an inevitable *consequence*. Now, the opinions advanced here to-night would indicate an exactly opposite state of influences. It is contended that the circulation in the arterial trunk supplying the gangrenous part has been arrested by ossification of the artery, or some other cause unconnected entirely with the part mortified, and that death of the part occurred in consequence.

Post-mortem examination of the arteries may reveal a condition which may be taken as the cause of the whole difficulty—such as ossification of their walls, occlusion of their calliber, from semi-organized fibrine, etc.; but it must be remembered that ossification is often found to exist when no such results follow, and that when the circulation in an artery is arrested from ligature or other cause, that from the point of obstruction toward the heart to the first bifercation the caliber is found obliterated by soft stricture.

The exact cause or causes operating in the arrest of nutrition resulting in gangrene is not always known. Derangement of nervous influences and an impoverished condition of the blood may at times account for the difficulty.

D*r. Johnson* agrees with the learned gentleman, Dr. Miller, that other causes than the ossification of an artery may produce the disease in question. Whatever impairs the quality of the blood, depriving it of its nutrient products, or whatever impairs the energy of the heart, with loss of expansive force in the large and contractile in the small arteries—thus depriving the capillaries of their due supply of nourisnment and the limb of its proper standard of warmth—may be set down as the true pathology of senile or chronic gangrene. But he desires to call attention to the treatment which has cured one, at least, of the cases, and will probably relieve both—viz., stimulants and nourishment, scrupulously preserving the warmth of the limb, with stimulating applications to the sore. Both subjects are negroes—one sixty-two, the other sixty-eight years old; in both, the temperaments cold and phlegmatic; both stingy and avaricious; both have the money to pay their bills. They have lived upon e least that human nature could subsist on since the war, the better to enable them to accumulate a reserve of cash.

*Dr. W. F. Westmoreland* reported a case of aneurism of the axillary artery, in which the aneurismal sack was opened and filled with charpie, saturated with muriated tincture of iron. The patient thus treated, some four weeks since, is doing well. No difficulty attended nor resulted from the operation, except a slight hemorrhage which occurred some twelve days after, from a small artery communicating with the wound. He preferred, in the case operated on, muriated tincture of iron for various reasons. The coagulation of blood and contraction of the blood-vessels and surrounding tissues are the direct influences of the remedy in arresting hemorrhage. In the case under consideration, the advantages derived from it are due to its irritating effect upon the sack and soft parts contiguous, causing the effusion of coagulable lymph, thereby promoting permanent adhesion of its walls and consequent security against return of the anurismal tumor. In his experiments with this preparation, some difficulty is found in the use of different specimens, owing, perhaps, to the improper mode of preparing it by some pharmacecutists.

*Dr. D. C. O'Keefe* thought it important to determine positively, by experiments on animals, whether the favorable result likely to be realized in the case reported be due to the mechanical influence of the charpie crowded into the anurismal sack, or to the peculiar styptic and irritating properties of the muriated tincture of iron.

Atlanta, December 3d, 1867.

*Dr. A. W. Griggs*, of West Point, being invited to meet with the Society, reported a case of recto-vaginal and vesico-vaginal fistulæ. No operation had been perfored nor any special treatment adopted; but, after the lapse of six or eight months, the opening from the vagina to the rectum closed spontaneously. The vesico-vaginal fistula continued, and in this condition the woman became pregnant. At her confinement in labor, it was ascertained that a tough fibrous growth, interspersed with calcarious deposit, near the external part of the vagina, interfered with the delivery. Without a free incision through this unyielding structure, the passage of the head through the vagina could not be effected. The sand-like substance throughout this abnormal growth made its division difficult. Finally, with a curved bistoury, the division was made and delivery effected.

*Dr. S. H. Stout* was of opinion that the grit-like substance found in the growth referred to was similar to the structure found in what are called ossified arteries. Pathologists deny the existence of true bony substance in such cases, and assert that, without calcarious deposition, this peculiar structure, with a low degree of vitality, only resembles in some respects bony structure.

*Dr. W. F. Westmoreland* thought it not improbable calcarious deposit really existed in the structure described by Dr. Griggs. He generally found such deposition in urinary fistulæ of long standing.

*Dr. J. G. Westmoreland* reported a case of obstinate facial and pericranial neuralgia, which, after cupping, the use of opium hypodermically and otherwise, quinine and mercurial cathartics, continued unabated. From the fact that opium, whether used by hypodermic injection or in the stomach, tenped rather to aggravate than lessen the pain, the idea was suggested that an action antagonistic to that of opium would probably be of service. Accordingly, the extract of belladonna, in doses of a fourth of a grain, was given every six hours. Relief was promptly afforded; and two days after, when the pain returned, the remedy was again administered with permanent success.

Atlanta, December 17th, 1867.

*Dr. J. M. Boring* reported a case of probable uterine disease, from which the woman suffered excessive pain in the hypogastric region, with constant nausea and vomiting. Was called at 7 o'clock A. M., and obtained the following history of the case: Six weeks previously she had menstruated properly, and up to the night before my visit, had been in good health. Was taken some four or five hours since with the symptoms before described. On examination, the abdomen presented considerable fulness just above the pubis, which was soft to the touch and very tender. She also complained of strangury. A combination of camphor, assafœtida and hyoscyamus was administered, without affording relief to the sufferer. Returning again at 2 o'clock, found the patient in a sinking condition, and prescribed brandy and chloroform, without benefit. The patient continued to sink, and died during the following night. The request for post-mortem examination was granted.

The opinion entertained by the reporter is that internal hemorrhage, probably from extra-uterine fœtation, led to the symptoms and fatal termination.

*Dr. Douglas* favored the probability of violent peritonitis being the cause of death. He thought it not impossible that this condition should lead to the symptoms in the case, and its rapid progress to a fatal termination.

*Dr. J. G. Westmoreland* did not think the time from the attack to the death of the patient was sufficiently long for death to have resulted from peritonitis, being less than twenty-four hours. On the other hand, it is not very likely that hemorrhage, to the extent of producing such violent symptoms, would result from two weeks' development of an impregnated ovum, whether extra-uterine or otherwise located. While these doubts as to the cause of hemorrhage are indulged, however, it must be confessed that the symptoms detailed are such as might possibly result from loss of blood in the pelvis.

*Dr. J. M. Johnson* thought it not unreasonable to suppose that fatal hemorrhage may occur from the cause assigned by Dr. Boring.

*Dr. J. G. Westmoreland* reported a case of strabismus in a child of four years old. Only one eye was affected, and the direction was obliquely inward. Complete relief was afforded in a few hours by applying a bandage over the nnaffected eye, so as to compel the child to use the affected eye alone.

